# Ultrasonic Manifestations of Mesenteric Inflammatory Myofibroblastic Tumors in Children

**DOI:** 10.3389/fped.2019.00039

**Published:** 2019-03-05

**Authors:** Jingjing Qian, Kun Zhu, Jingjing Ye

**Affiliations:** ^1^Department of Ultrasound, Children's Hospital, Zhejiang University School of Medicine, Hangzhou, China; ^2^Department of Pathology, The Children's Hospital, Zhejiang University School of Medicine, Hangzhou, China

**Keywords:** children, inflammatory myofibroblastic tumor (IMT), ultrasonic manifestations, mesentery, computed tomography (CT)

## Abstract

**Objective:** To explore the ultrasonic manifestations of mesenteric inflammatory myofibroblastic tumors (IMTs) in children.

**Methods:** Seven patients with mesenteric IMTs were retrospectively analyzed. The ultrasonic manifestations, such as the locations, sizes, morphology, borders, internal echo, blood flow, and metastasis, of the tumors were detected.

**Results:** In all the seven pediatric patients, a solitary lesion was found for the mesenteric IMTs, including five cases in the ileocecal mesentery and two cases in the mesentery of ascending colon. All the single tumors were revealed as irregular hypoechoic masses with uneven internal echoes and enhanced echoes in the surrounding intestine and omentum. Internal blood flow signals were enriched in the tumors. The borders were clear in five cases and unclear in two cases. In addition, two cases had peritoneal effusion and one case had calcified plaques. In the follow-up studies, one of the seven IMT patients had malignant transformation, and one case was transferred to the pelvic cavity.

**Conclusion:** Ultrasonic examination can clearly demonstrate the locations, sizes, morphology, borders, internal echo, blood flow as well as metastasis of the pediatric IMT of the mesentery, having an important clinical application value as an adjunct to computed tomography (CT).

## Introduction

Inflammatory myofibroblastic tumors (IMTs), also known as inflammatory fibrosarcomas and inflammatory pseudotumors are composed of myofibroblastic spindle cells accompanied by an infiltrate of variable numbers of inflammatory cells including plasma cells, lymphocytes, immunoblasts, eosinophils, and fibrous tissue (1, 2). These tumors are uncommon mesenchymal tumors that preferentially occur in children and adolescents (3). IMTs can usually occur throughout the body, and mesentery and omentum are the most commonly involved sites apart from lungs (2, 4–6). Although the etiopathogenesis of IMTs is poorly understood, various factors such as human immunodeficiency virus infection (7), granulations (8), trauma, surgery, and neoplastic mechanisms are considered to be related with IMTs (4, 9, 10). Total resection of tumors is usually performed to treat IMTs (5). And ultrasound and computed tomography (CT) can be commonly used as the auxiliary examinations for confirming the diagnosis of the tumors. Ultrasound has a strong advantage in observing the activities of the tumors (11). However, previous reports on the ultrasound of pediatric abdominal IMT mostly focused on individual cases (12).

In the present study, we retrospectively analyzed the ultrasound images of 7 pediatric patients with mesenteric IMT combined with the patients' clinical medical history and previous studies, providing a better understanding of IMTs, as well as indicating ultrasound as an effective method for the preoperative diagnosis and long-term follow-up studies of pediatric IMT of the mesentery.

## Materials and Methods

### Patients

A total of seven pediatric patients pathologically diagnosed with inflammatory myofibroblastic tumors (IMTs) and admitted to Children's Hospital, Zhejiang University School of Medicine (Hangzhou, China) between May 2010 and March 2017 were enrolled after obtaining the consent of the patient and family. All the seven patients received ultrasound examinations, which were performed prior to the operation. This study only involved a retrospective review of the imaging data.

### Instruments and Methods

Philips iU Elite color ultrasonic diagnostic apparatus with convex array probe (1–5 MHz) and linear array probe (5–12 MHz) was purchased from Philips Healthcare (Eindhoven, NL) and used in this study. The pediatric patients were placed in a supine position to fully expose the abdomen throughout the examination. The patients who didn't cooperate were sedated with 10% chloral hydrate by enema. Firstly, variables including tumor locations, sizes, morphology, borders, relation to surrounding tissues, and presence of metastasis were scanned using the convex array probe. Afterward, variables such as tumor internal echo characteristics, presence or absence of calcification or liquefaction, and changes in blood flow signals and surrounding tissue echo were observed carefully using the linear array probe. All in all, the patients' imaging data were collected and analyzed. After treatment, the presence or absence of postoperative recurrence and metastasis have also been recorded. The children were followed up in the clinic at 1, 3, 6, and 12 months after surgery and reviewed for ultrasound. For children with distant metastases, CT or magnetic resonance imaging (MRI) was performed in addition to the ultrasound examination.

## Results

### Clinical Information

The general information was showed in Table 1. The patients consisted of five males and two females, aged from 3.8 to15 years with an average age of 8.8 years. In this study, all the patients showed clinical symptoms, including three patients with abdominal pain, one patient with an abdominal mass, four patients with fever, one patient with wheezing sensation accompanied by vomiting after eating for more than one year, one patient with frequent micturition, the urgency of urination, and the dysuria, as well as one patient with concurrent intussusception. Among these cases, one patient had a history of abdominal trauma due to the traffic accident, and another one had a history of an appendicular abscess.

**Table 1:** Patient demographics and clinical features.

**Case number**	**Gender**	**Age**	**Clinical symptom**	**Ultrasound manifestations**	**Primary site**	**Metastasis**	**Treatment**	**Follow-up**	**Recurrence**	**Survival**
1	Male	4y3m	Liver contusion 6 months ago	A low echo mass of about 9.1 * 7.5 * 5.3 cm was detected in the lower right abdomen, with an irregular shape; there were a nodular echo inside and more abundant blood flow signal	Ascending colon wall	No	Surgical resection	3 months	No	Yes
2	Female	3y10m	Abdominal pain for 2 days	A hypoechoic mass of about 8.5 * 6.2 * 5.7 cm was probed in the lower right abdomen, with an irregular shape; there were nodular and hemp-like echoes and more abundant blood flow signal	Colonic serosa	No	Surgical resection plus chemotherapy	3 years	No	Yes
3	Male	14y8m	Frequent micturition, the urgency of urination, and the dysuria for half a month	A hypoechoic mass of about 6.3 * 5.4 * 5.0 cm was detected in the upper anterior bladder, with an irregular shape; there were nodular and hemp-like echoes and more abundant blood flow signal	Posterior wall of the bladder	Pathology: malignant	Surgical resection and chemotherapy	3 years	No	Yes
4	Male	14y9m	After eating, wheezing sensation accompanied by vomiting for more than 1 year, which was aggravated for 3 months	During chemotherapy, a low echo mass of about 2.7 * 2.2 * 2.1 cm was seen in the upper left of the bladder; the shape was irregular, the echo was not uniform, and there were nodular and hemp-like echoes inside and more abundant blood flow signal	Gastric-momentum	Pelvic cavity	A gastric, omental mass was found by gastroscope, CT, and small intestine water imaging (MRI), followed by surgical resection and chemotherapy; during the chemotherapy, a mass in the pelvic cavity was detected by ultrasound; then surgical resection and chemotherapy were performed	3 years	No	Yes
5	Female	4y4m	Abdominal mass with intussusception	A mass of 4.3 * 3.2 * 2.7 cm was found after right mid-abdominal exploration; the transverse section was concentric circle and the longitudinal section was pseudonephrogram; no bowel movements were seen (the above was an intussusception image); a low echo envelope of 3.6 * 3.3 * 3.2 cm can be detected in the inner side (upper right abdomen) with an irregular shape; the boundary was still clear, the internal echo was still uniform, and rich blood flow signal can be detected in the low echo envelope	Ascending colonic mucosa	No	Surgical resection	6 months	No	Yes
6	Male	15y	Metastatic right lower abdominal pain for 3 days	A low echo mass of about 7.3 * 3.9 * 6.4 cm was detected in the lower right abdomen, with an irregular shape; the boundary was unclear; the internal echo was uneven; the calcified plaque with a diameter of 1.0 cm can be seen inside; the echo of the surrounding omentum was enhanced; and the the rich blood flow signal was explored	Right lower quadrant	No	Biopsy and automatic discharge after anti-inflammatory treatment, without resection	Lost		
7	Male	4y8m	Intermittent abdominal pain for 1 week	A low echo mass of about 6.4 * 6.3 * 5.6 cm was observed in the right middle lower abdomen, with an irregular shape; the boundary was unclear; the internal echo was uneven; the echo of the surrounding omentum was enhanced; and the rich blood flow signal was explored	Colonic serosa	No	Surgical resection	8 months	No	Yes

Based on the location of the primary lesion, the feasibility of the complete removal of tumor by surgery, and the occurrence of a metastasis, the use of chemotherapy was determined. If the tumor was confined to the primary site, which can be completely removed by surgery, no chemotherapy was needed; if the tumor cannot be completely removed, with surrounding tissues involved, malignant changes, or distant metastases, the chemotherapy was required (Table 1).

### Ultrasound Manifestations

**Figure 1:**
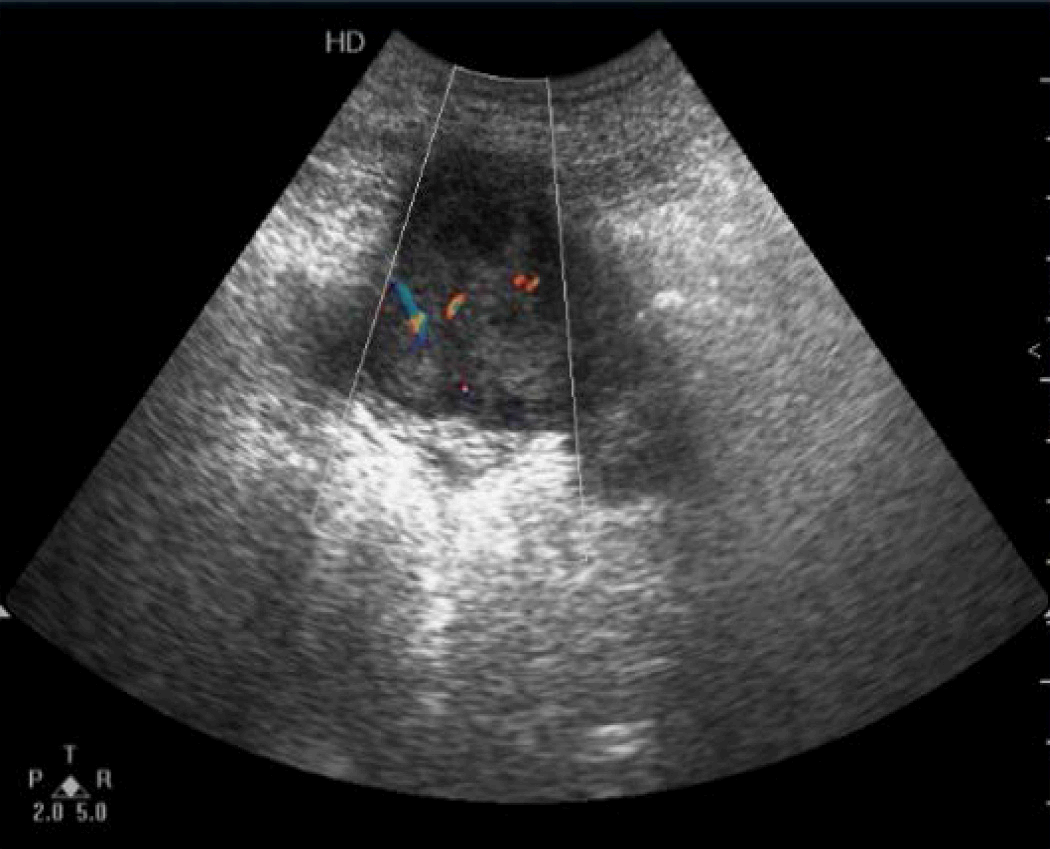
Detection of the right lower abdomen. Irregular hypoechoic mass which showed lobulated enhanced echoes in the surrounding intestine and omentum, and enriched internal blood flow signals within the mass.

**Figure 2:**
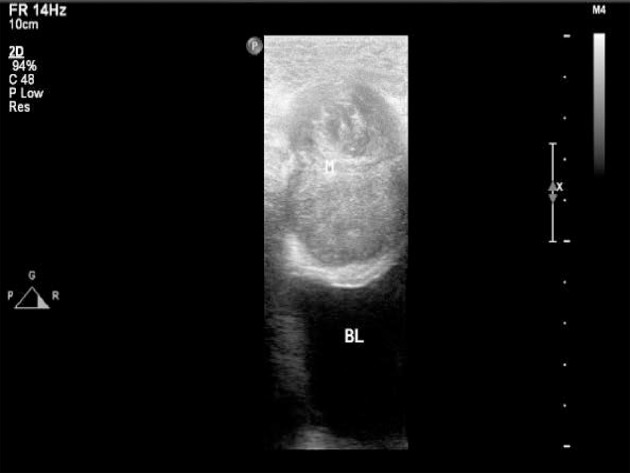
High-frequency ultrasound showed irregular shape, uniform echo, and nodular and hemp-like echoes inside.

**Figure 3:**
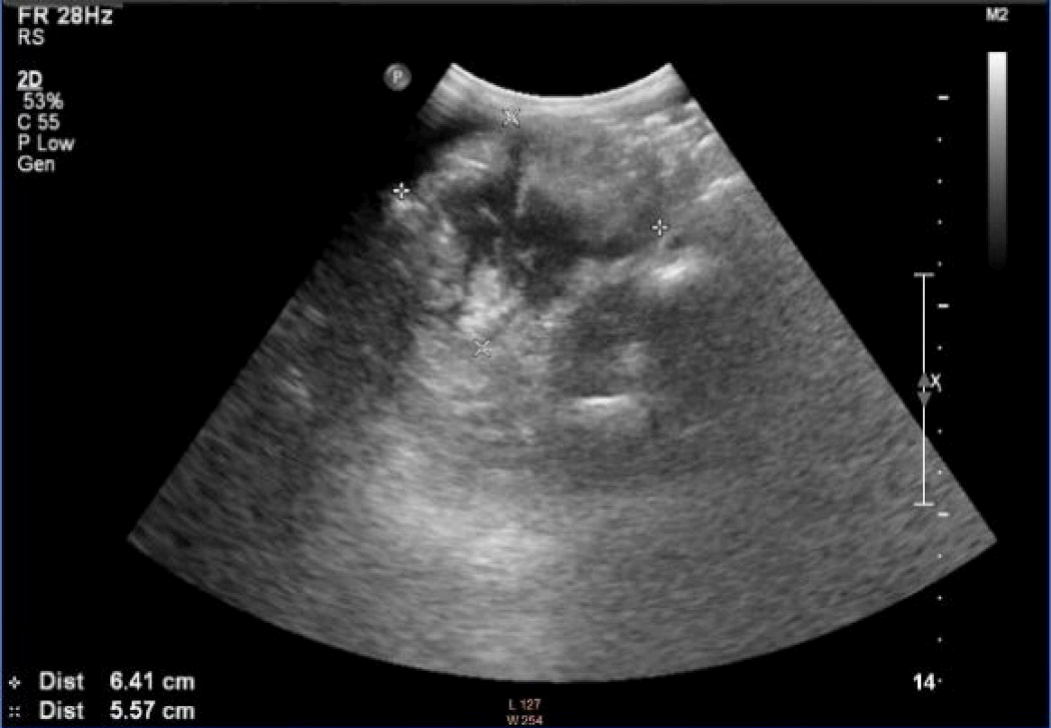
Detection of the right lower abdomen. Irregular hypoechoic mass with uneven internal echo and enhanced echoes in surrounding intestine and omentum required the distinguishment from an appendicular abscess.

**Figure 4:**
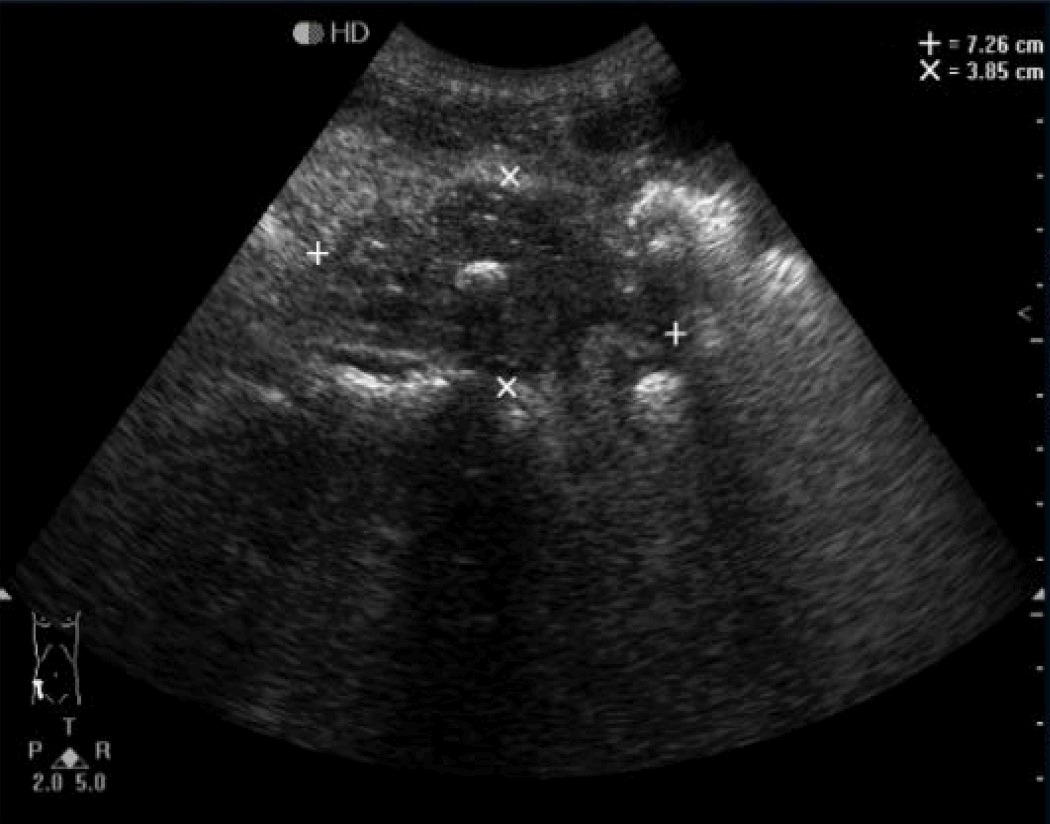
Detection of the right lower abdomen. Irregular hypoechoic mass with uneven internal echo was detected in the right lower abdomen. Bright echo spots could also be visible.

We then analyzed the sonograms of seven patients with mesenteric IMTs and the results are showed in Table 1. In all the seven pediatric patients, a solitary lesion was found for the mesenteric IMTs, including five cases in the ileocecal mesentery and two cases in the mesentery of ascending colon. The minimum size of the tumor detected by ultrasound was 2.7 ^*^ 2.2 ^*^ 2.1 cm. We found that the maximum average diameter was 6.3 cm. And the tumors had been shown as irregular hypoechoic masses with uneven internal echoes in all patients (Figures 1, 2). In addition, the echoes had been enhanced in the surrounding intestine and omentum, and the internal blood flow signals within the masses had also been enriched in all patients (Figures 1, 3). Furthermore, five out of the seven masses showed an irregular shape with nodular, clump-like, or hemp-like echoes inside (Figures 1, 2). These should be distinguished from common pediatric abdominal lipoma and lymphoma. Meanwhile, two out of the seven masses showed irregular shape and unclear borders, the uneven internal echoes, the enhanced echo of the surrounding omentum, and the rich internal blood flow signal, which had to be distinguished from the abdominal inflammatory masses of appendicular abscess commonly found in children (Figures 3, 4). Among the seven IMT patients, two and one patients were accompanied by peritoneal effusion and calcified plaques, respectively (Figure 4).

As mentioned above, the preoperative ultrasonography showed that two of the pediatric patients had inflammatory masses, indicated by the irregular shape, unclear borders, uneven internal echoes, enhanced echo of the surrounding omentum, and rich internal blood flow signal. This finding was confirmed by their laboratory results, where increased levels of white blood cell (WBC) and C-reactive protein (CRP) were found in the four patients (data not shown).

Notably, except for the 4y4m female patient who underwent ultrasound, the other six patients underwent CT and magnetic resonance imaging (MRI) before and after surgery. The ultrasound results for the location of the mass, the measurement of the size, the determination of the boundary, the relationship with the surrounding tissue, and the presence or absence of distant metastases in the abdomen were most consistent with the results from CT and MRI. But ultrasound is easier to operate, especially for children, and it is more reproducible than CT and MRI. In the two cases that the mass and appendix abscess were difficult to distinguish in the ultrasound image, the inflammatory mass was also considered in the contrast-enhanced CT scan.

### Follow-Up

The patients were followed up at 1, 3, 6, and 12 months after surgery. The follow-up duration was 8 (3–36) months. One patient was lost in the follow-up. Ultrasound was the main means of re-examination. CT or MRI was performed in patients undergoing chemotherapy or distant metastasis. One of the seven IMT patients had malignant transformation, one case was transferred to the pelvic cavity, and no recurrence was observed (Table 1). As the tumor was removed, the clinical symptoms disappeared and the inflammatory blood tests were normal.

## Discussion

IMTs are relatively uncommon soft tissue lesions. Brunn described them as lung spindle cell carcinoma in two patients in 1939 for the first time (13), and IMT was referred to as inflammatory pseudotumor (IPT) or plasma cell granuloma (PCG) at that time. Afterward, this disease was named as IMT according to the WHO classification of soft tissue tumors in 1994, which referred to a type of tumor comprised of differentiated myofibroblastic spindle cells accompanied by large amounts of plasma cells and/or lymphocyte infiltration. IMT was classified as an intermediate type of tumor due to the potential of recurrence and distant metastasis (14). IMTs can occur throughout the body and are often found in the mesentery and retroperitoneum of children and adolescents (15). In the present study, all the seven pediatric patients were diagnosed with mesenteric IMTs, with the ileocecum and ascending colon as the most common sites for the tumor development.

The pathogenesis of IMT is still controversial. Studies have confirmed the gene rearrangement and expression of anaplastic lymphoma kinase (ALK) in 45–50% IMTs (16, 17). Since the IMTs are mainly composed of myofibroblasts that participate in the growth, repair and scar formation of normal tissues (18), it is hypothesized that the IMTs may result from the overreaction of myofibroblasts induced abnormal repair following surgery, trauma, or inflammatory lesions (19, 20). Besides, some studies also indicated that IMTs were associated with the infection of human herpesvirus type 8 (HHV-8) and Epstein-Barr virus (EBV) (21, 22). However, IMTs have been shown to be related to EBV and HHV-8 in only a few cases (23). In our study, one case had a history of abdominal trauma due to the traffic accident, and another one had a history of the appendicular abscess. Therefore, the IMTs in these two cases may result from the overreaction of myofibroblasts induced abnormal repair. However, the further studies of the pathogenesis of IMT are still required.

As above mentioned, most IMTs are the potentially malignant intermediate type of tumors (24), often presenting a benign clinical course with the possibility of metastasis and recurrence (25). The recurrence rates of pulmonary and extra-pulmonary IMTs were between 2 and 25%, and the incidence of distant metastasis was <5% (26). In the present study, one of the seven IMT patients had malignant transformation, one case was transferred to the pelvic cavity, and no recurrence was observed in the follow-up studies. The onset of IMT is relatively unnoticeable, and its clinical symptoms depend on the location of development, mostly caused by the mass itself and the tumor's compression on the surrounding visceral organs (26). About 15–30% of patients with IMTs are accompanied by the systemic symptoms such as fever, pain, tiredness, night sweats, and weight loss. Although laboratory examinations can show the signs of microcytic anemia, thrombocytopenia, and elevated erythrocyte sedimentation, these signs lack specificity and tend to disappear frequently following tumor excision (14). In our study, all the seven pediatric patients with IMTs presented the clinical symptoms, including three cases with abdominal pain, one case with abdominal mass, four cases with fever, one patient with wheezing sensation accompanied by vomiting after eating for more than one year, which was aggravated for three months, one case with frequent micturition, the urgency of urination, and the dysuria, as well as one case with concurrent intussusception. In addition, according to laboratory studies, four out of the seven patients showed increased levels of WBC and CRP, indicating the occurrence of inflammation.

The imaging examinations of IMTs lack specificity due to the different development sites and histopathological diversity of tumors (27, 28). Although CT and MRI are the most commonly used imaging methods in the evaluation of IMTs, ultrasound is usually the first choice when IMTs are found in specific regions, such as testicles or neck (29). As reported, ultrasound is the most accurate imaging approach in the evaluation of scrotal masses (30). For the examination of mesenteric IMTs, CT is usually used. For children with abdominal disease, ultrasound is the preferred method of examination, and CT and MRI are performed after ultrasound. However, previous reports on the ultrasound of pediatric abdominal IMT mostly focused on individual cases, leading to diverse and less specific imaging features of IMTs. In the present study, we analyzed the sonograms of seven patients with mesenteric IMTs combined with their medical history, trying to figure out whether ultrasound could be used as a complement for CT in the preoperative diagnosis and long-term follow-up studies of pediatric IMT of the mesentery. We found that the ultrasound examination of the lower right abdomen showed an irregular hypoechoic mass that was partially lobulated, with uneven internal echo (12). Besides, the masses showed enhanced echoes in the surrounding intestine and omentum, and enriched internal blood flow signals (Figures 1, 2). Further, the two of seven cases showed the irregular shape, the unclear boundary, the uneven internal echo, and the enhanced echo of the surrounding omentum, which had to be distinguished from the abdominal inflammatory masses of appendicular abscess commonly found in children (Figure 3). The enriched internal blood flow signals within the masses were the main point of the differential of IMTs. On the other hand, the remaining five masses that showed an irregular shape with nodular and clump-like echoes inside and there out of the seven masses showed hemp-like echoes inside, which had to be distinguished from common pediatric abdominal lipoma and lymphoma. The ultrasonic manifestations of lipoma are presented as moderate echo or hypoechoic with homogeneous internal echo, and lymphoma is most often shown as very hypoechoic approximate to echoless mass with clear border (31). Calcification and hemorrhagic necrosis are rarely detected in IMTs (3), and calcification was found in only one out of the IMT patients without any detectable echoless dark regions of the tumor mass in the present study.

As above mentioned, IMT is a benign tumor that has been mainly caused by the proliferation of myofibroblasts at a basic level of inflammation. Considering that the preoperative diagnosis of IMT by ultrasound alone is not reliable, histopathological, and immunohistochemical examinations are required for the definitive diagnosis of IMT. In the present study, the ultrasound images revealed the signs of inflammatory changes in four pediatric patients with mesenteric IMT. After taking the clinical history into account, the patients subsequently received anti-inflammatory treatments. Then repeated ultrasound reexaminations were performed. The possibility of IMT should be considered when no significant tumor reduction or tumor enlargement was indicated by ultrasound examination. Since ultrasound is inexpensive, convenient, non-invasive, non-radioactive and reproducible, and provides successive dynamic observation on lesions, it plays an important role in the preoperative diagnosis of pediatric IMTs and thus has an important clinical application value.

There are several limitations of this study. First, this is a single-center study. Second, the sample size is small. Third, no comparison has been made between ultrasound and other imaging methods such as MRI or CT in this study. Fourth, the role of the ultrasound in identifying relapsed IMTs was not possible in this study as none of the seven patients had any recurrence. Lastly, ultrasound is operator-dependent, which is the biggest limitation of this study. Large-scale multi-center studies will be performed to confirm our findings in the future.

In conclusion, the ultrasonic examination has important medical applications in the preoperative diagnosis and postoperative follow-up studies of pediatric IMTs of the mesentery, being an important adjunct to CT abdomen and providing a better understanding and enhanced diagnostic capacity of IMTs.

## Ethics Statement

This retrospective study was approved by the Ethics Committee of Children's Hospital, Zhejiang University School of Medicine (Hangzhou, China). All subjects gave written informed consent in accordance with the Declaration of Helsinki.

## Author Contributions

JQ conceived and designed the study, performed the examination, collected and analyzed the data, and drafted the manuscript. KZ helped to collect and analyze the data. JY helped to conceive of the study and perform the examination. All authors read and approved the final manuscript.

### Conflict of Interest Statement

The authors declare that the research was conducted in the absence of any commercial or financial relationships that could be construed as a potential conflict of interest.
